# Levamisole-Adulterated Cocaine: A Case of Vasculitis and Severe Neutropenia

**DOI:** 10.7759/cureus.100358

**Published:** 2025-12-29

**Authors:** Bartholomew Olash

**Affiliations:** 1 Internal Medicine, Rush University Medical Center, Chicago, USA

**Keywords:** agranulocytosis, anca-associated vasculitis, cocaine, levamisole, severe neutropenia

## Abstract

Levamisole is an immunomodulatory agent that was formerly used in the United States as a disease-modifying antirheumatic drug (DMARD) for rheumatoid arthritis (RA). More recently, it has become a common adulterant in cocaine. Levamisole exposure has been associated with a broad spectrum of adverse effects, such as neutropenia, agranulocytosis, distinctive skin lesions, vasculitis, arthralgias, and leukoencephalopathy. While the literature on complications related to levamisole exposure is currently sparse, given its increasingly common use as a cutting agent in cocaine, it is likely that they are underreported. Because of this, heightened vigilance among clinicians is warranted. Here we present an unusual case of levamisole toxicity causing both vasculitis with systemic and cutaneous pathology, including severe neutropenia complicated by septic shock.

## Introduction

Levamisole is an immunomodulatory agent previously used in the United States for rheumatoid arthritis (RA). It was taken off the market in the U.S. in 2000, in large part due to its significant and potentially severe side-effect profile, most notably vasculitis and agranulocytosis [1]. However, it remains in use as an antihelminthic in veterinary medicine and is the most common cutting agent found in cocaine [2, 3].

Reports of levamisole toxicity remain quite rare. However, given the ubiquity of cocaine use in the United States, with approximately 41 million individuals reporting lifetime use, it is important for clinicians to maintain familiarity with both common and uncommon pathologies associated with its use [4].

As previously mentioned, toxicity from levamisole can manifest in a range of serious clinical symptoms. It is well documented to cause severe neutropenia and agranulocytosis, both of which can be life-threatening [1, 5]. Other manifestations include leukoencephalopathy and antineutrophil cytoplasmic antibody (ANCA)-mediated vasculitis, which has been reported to result in nasopharyngeal, pulmonary, and renal pathology, along with characteristic purpuric skin lesions that may ulcerate [1, 6, 7].

This case is a relatively unique presentation of levamisole ingestion, as it presents a patient with multisystem pathology, much of which is most likely directly attributable to levamisole ingestion via vasculitis and severe neutropenia leading to septic shock.

## Case presentation

A middle-aged woman with a history of RA, hypothyroidism, and cocaine use disorder presented to the emergency department with generalized weakness and a recent fall. Her additional complaints included chronic joint pain and a new rash on her face and lower extremities.

**Figure 1:**
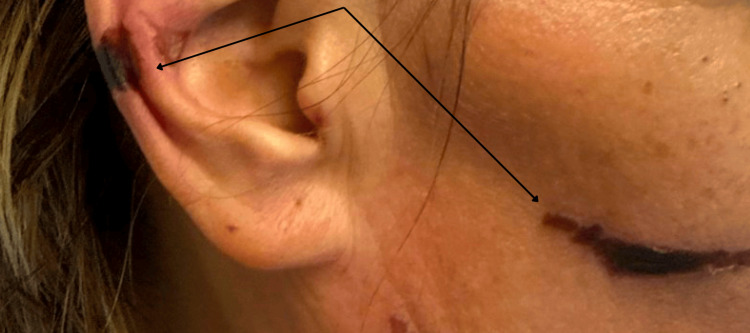
Retiform purpura of the helix of the ear The arrow demonstrates an example of non-blanching, purpuric lesions on the helix of the patient's ear and face.

**Figure 2:**
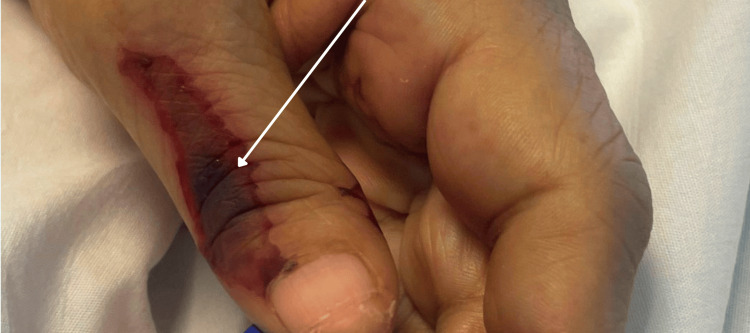
Retiform purpura of the left first digit The arrow demonstrates an example of non-blanching, purpuric lesions present on the patient's face, trunk, and extremities

Upon admission, she was tachycardic and tachypneic but normotensive. Notable findings included fine bibasilar crackles bilaterally, ulnar deviation, synovitis, joint tenderness at the wrists and metacarpophalangeal joints, and violaceous lesions on her cheeks, ears, fingers, and lower extremities. Examples of her skin lesions are shown below in Figures 1, 2. She also exhibited significant voice hoarseness and hypophonia.

**Figure 3:**
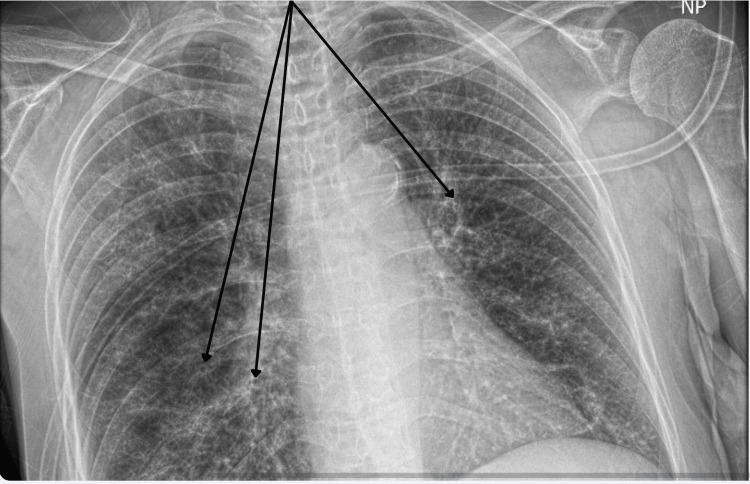
Initial Chest Radiograph The arrows demonstrate examples of the described diffuse bronchial wall thickening.

The initial complete blood count showed mild pancytopenia and mild neutropenia, as demonstrated in Table 1. Her white blood cell and neutrophil counts further declined after admission, then recovered spontaneously, as shown in Table 2. Additional tests on admission included a CT of the head, which was negative for acute abnormality, and a chest X-ray re-demonstrating previously noted diffuse bronchial wall thickening, shown in Figure 3.

**Table 1:** Initial Complete Blood Count (CBC) The CBC demonstrates pancytopenia present on admission.

Component	Reference Range	Patient Value
White blood cells (K/uL)	4-10	2.03
Red blood cells (M/uL)	4-5.2	3.23
Hemoglobin (g/dL)	12-16	8.6
Hematocrit (%)	37-47	26.2
Mean corpuscular volume (fL)	82-103	81.1
Mean corpuscular hemoglobin (pg)	26-34	26.6
Mean corpuscular hemoglobin concentration (g/dL)	30-37	32.8
Red cell distribution width (g/dL)	11.5-14.5	17.7
Platelet count (K/uL)	150-399	58

**Table 2:** Neutrophil and White Blood Cell Trend Trend of white blood cell and absolute neutrophil count with nadir at day two and day three, respectively, followed by spontaneous recovery.

Component	Reference Range	Day 1	Day 2	Day 3	Day 4	Day 5
White blood cells (K/uL)	4-10	2.03	0.79	0.87	1.80	8.43
Neutrophils (K/Ul)	1.84-7.8	1.10	0.19	0.10	0.73	6.74

Initially, given evidence of RA flare as well as pancytopenia, rheumatology and hematology were consulted. She was treated with prednisone for her suspected RA flare. Initial work-up for pancytopenia, including Epstein-Barr virus, cytomegalovirus, parvovirus, *Helicobacter pylori*, hepatitis viral panel, HIV, folate, B12, zinc, copper, peripheral smear, and flow cytometry, was relatively unrevealing, showing only low folate at 4.8 ng/mL, which was repleted. Of note, she had been prescribed methotrexate, but the serum level was undetectable, and she was unsure when she had last taken it. Given this unrevealing work-up in the setting of her purpuric skin lesions and urine drug screen, which was positive for cocaine, dermatology and toxicology were consulted due to concern for levamisole intoxication. Levamisole urine studies obtained on day two of admission were negative. Atypical p-ANCA was elevated at 1:160 with c-ANCA within normal limits. Myeloperoxidase and proteinase 3 antibodies were also negative. ESR and CRP were both elevated at 78 and 49.1, respectively. Of note, the exact date of the patient's last cocaine use was unclear; the patient's daughter noted cocaine was present in her home during the week prior to admission, which is consistent with a positive assay with a window of detection of three to five days. 

**Figure 4:**
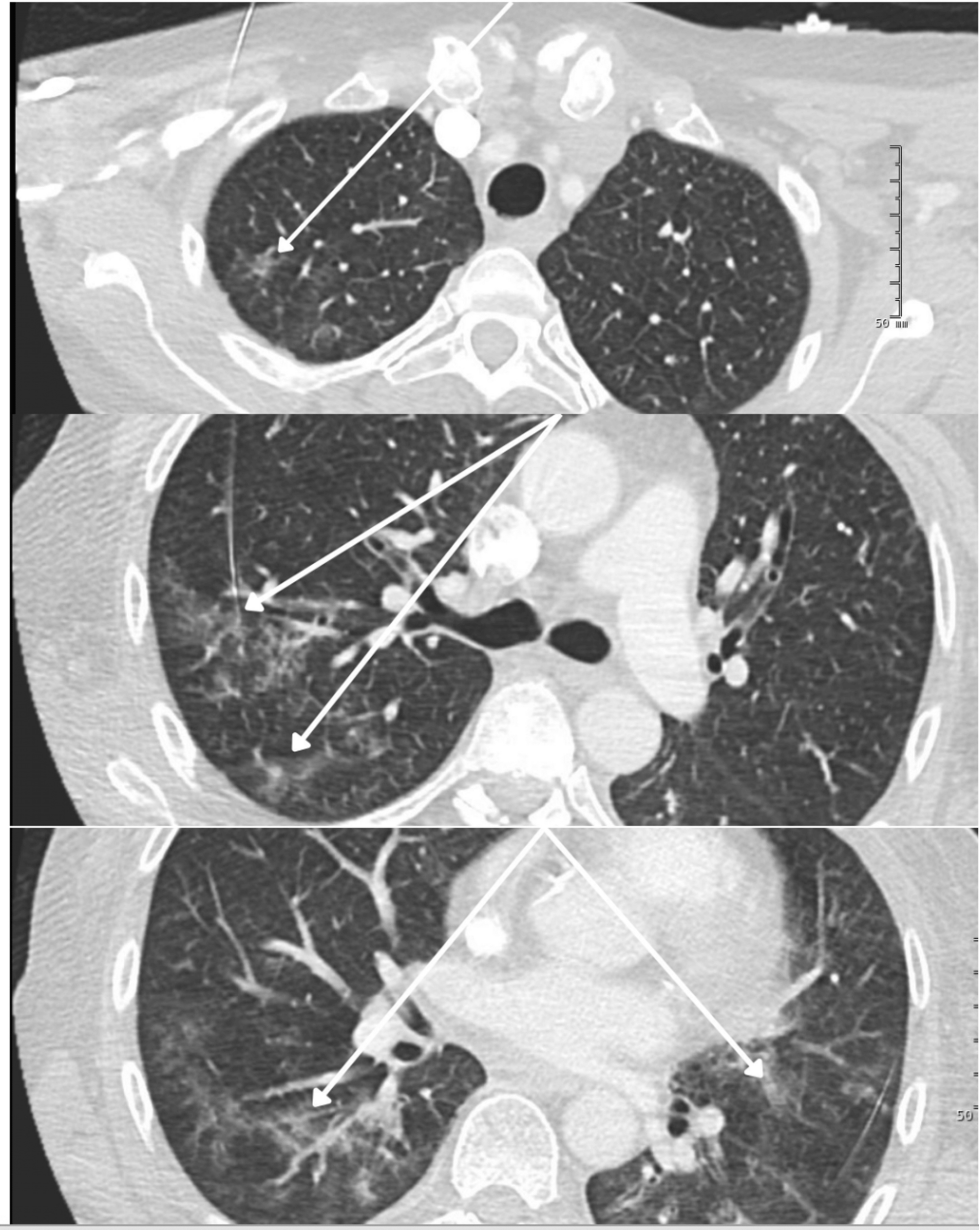
Selected Cuts of the CT Angiogram of the Chest Multifocal, bilateral ground glass opacities are demonstrated, more prominent on the right than the left, which were noted in all five lobes of the lung.

The patient’s hospital course was complicated by admission to the ICU for acute hypoxemic respiratory failure and the development of altered mental status. CT angiography of the chest was performed to rule out pulmonary embolus, which showed ground glass opacities diffusely throughout the lung fields bilaterally, as demonstrated in Figure 4. The patient was treated empirically for hospital-acquired pneumonia. An MRI of the brain was also performed in the setting of a new altered mental status, which did not demonstrate any acute intracranial abnormality or abnormal enhancement. She underwent bronchoscopy; however, all associated studies, including aerobic and anaerobic cultures, fungal smear and culture, legionella culture, viral panel, and pneumocystis stain, remained negative. She remained obtunded for several days and was treated empirically for meningitis; however, cerebrospinal fluid (CSF) studies obtained prior to initiation of treatment, including cultures, cell count, protein, glucose, and an autoimmune encephalitis panel, were also unrevealing.

By day 5 of hospitalization, her leukopenia and neutropenia had resolved. Mentation and acute hypoxic respiratory failure improved over the next several days. Speech language therapy was consulted for significant hoarseness, hypophonia, and suspected aspiration. Fiberoptic endoscopic swallow evaluation was performed, but the patient failed due to diffuse oropharyngeal edema. Her ICU course was complicated by the development of septic arthritis of the right hip with concomitant methicillin-resistant *Staphylococcus aureus* (MRSA) abscess formation along the iliacus muscle, which was treated with aspiration and vancomycin. Surgical intervention for her septic arthritis was deferred inpatient with ongoing planning for a two-stage outpatient repair.

## Discussion

Levamisole, once used as an anti-rheumatic agent and currently the most prevalent cocaine contaminant according to Drug Enforcement Administration (DEA) reports, is responsible for a wide range of adverse effects [3]. Toxicity has been linked to agranulocytosis, severe neutropenia, and ANCA-associated vasculitis [2, 3]. This case demonstrates suspected levamisole toxicity causing both ANCA-associated vasculitis and severe neutropenia, complicated by septic arthritis and iliacus abscess formation.

The most characteristic finding of levamisole toxicity in this case was the patient’s retiform purpura, shown in Figures 1, 2. These purpuric lesions may become necrotic. The skin lesions and many other associated adverse effects are attributable to ANCA-mediated vasculitis. While the pathophysiology is not completely understood, the clinical and laboratory features resemble other ANCA-associated vasculitides. One distinguishing feature is very high atypical p-ANCA titers due to human neutrophil elastase, which was also present in this patient [8]. Although her urine levamisole assay was negative on day two of admission, this is not unexpected given levamisole’s short half-life (~3 hours) [9]. Unless testing is conducted promptly, results are likely to be negative. It is important to note that cutaneous vasculitis can rarely be caused by an RA flare. However, this patient's recent cocaine use as well as her high atypical p-ANCA titer are more suggestive of levamisole use, especially given the specific retiform pattern demonstrated above. Though skin biopsy was considered by dermatology, it was deferred given stable lesions, high suspicion for levamisole toxicity given its retiform appearance and distribution over the helix of the ears, and the patient's overall clinical deterioration requiring admission to the ICU.

Her acute hypoxemic respiratory failure was treated as hospital-acquired pneumonia in the setting of possible aspiration. However, levamisole has been associated with edema and necrosis of the upper respiratory tract, vocal cords, and lungs [1, 7]. While diffuse alveolar hemorrhage is most typical of ANCA-associated vasculitis, the diffuse, bilateral ground-glass opacities, including in the lingula, were less consistent with aspiration pneumonia. Given the extensive negative infectious workup and absence of focal consolidations, bacterial and fungal causes seem unlikely. Viral pneumonia in the setting of severe neutropenia was considered, but her respiratory pathogen panels, collected both from bronchoscopy and nasopharyngeal swab, were negative. Overall, given her extensive negative infectious work-up, diffuse bilateral ground-glass opacities (including in the upper lobes of the bilateral lungs), and upper airway edema causing hoarseness, infectious etiologies of her acute hypoxemic respiratory failure were considered unlikely. ANCA-associated vasculitis secondary to levamisole ingestion was thought to be more likely responsible for her respiratory compromise. Though the more typically described pulmonary-renal syndrome and diffuse alveolar hemorrhage were not present in this case, the presentation could be caused by a milder pulmonary capillaritis. Of note, her hemoglobin and hematocrit remained stable throughout her admission. Her dysphagia and significant upper airway edema could also be attributed to levamisole, as similar upper airway and vocal cord involvement has been reported, though the cases reported in the literature are more severe [1, 7].

The etiology of her prolonged altered mental status was less clear. Given an extensive negative work-up, including CSF, autoimmune, and paraneoplastic studies, meningitis and autoimmune encephalitis seem unlikely. The possibility of critical illness and septic shock contributing to her encephalopathy was considered. Although levamisole-induced encephalopathy is described in the literature [1, 6], the characteristic hyperintense T2 lesions and diffusion restriction expected in levamisole-induced leukoencephalopathy were not present on this patient’s MRI brain. Of note, the patient was not fully oriented on admission, and while mentation improved prior to discharge, her mental baseline was also unclear.

Another aspect very characteristic of levamisole toxicity was the severe, transient neutropenia and leukopenia. She presented with mild neutropenia, which rapidly progressed to severe neutropenia and then spontaneously recovered over four days. Of note, the patient did have a folate level of 4.8 ng/mL, which likely contributed to her chronic cytopenias. It is very unlikely, however, that her folic acid deficiency would cause an acute, severe neutropenia that completely recovered only three days after initiation of folate supplementation. Although methotrexate itself can cause neutropenia and leukopenia, the patient's methotrexate level on admission was undetectable, making it unlikely to be the causative agent. While there is no well-defined timeline for levamisole-induced agranulocytosis, a case series of six patients with confirmed levamisole exposure reported a median recovery time of six days (range: one to 20 days) [10]. 

Despite its brief course, the patient developed septic arthritis with an MRSA abscess along the iliacus muscle as a result of her severe neutropenia. The previously mentioned case series found a high complication rate, with most patients developing severe infections and several requiring ICU admission [10]. Individuals with repeated exposure to contaminated cocaine may be at particularly high risk for severe, invasive infections, and clinicians should maintain a high index of suspicion for infectious complications in the setting of clinical deterioration.

Overall, this case demonstrates the potential for multisystem involvement and significant infectious complications in levamisole-associated ANCA-mediated vasculitis and neutropenia. While skin findings, severe neutropenia, and specific organ-related complications of ANCA-mediated vasculitis have been discussed in other cases, the concomitant development of both severe neutropenia with eventual septic shock and signs of a mild but still clinically significant ANCA-mediated vasculitis in the lungs, upper airway, and skin highlights the potential for significant variety in both the severity and presentation of levamisole-associated toxicity. While the skin lesions, ANCA pattern, and spontaneous recovery of neutropenia and hypoxemia with lack of any other identifiable cause in the setting of known cocaine use strongly support levamisole-associated toxicity, an important limitation of this report is that levamisole was not identified in the urine, as discussed above.

## Conclusions

Cocaine is commonly abused in the United States and is frequently adulterated with substances such as levamisole, which, according to current DEA data, is the most prevalent contaminant. As discussed, levamisole toxicity may lead to a wide array of clinical presentations due to its immunomodulatory effects. It is essential for clinicians to recognize its potential to cause life-threatening neutropenia, agranulocytosis, and severe manifestations of ANCA-associated vasculitis. Given the potential for repeated exposure to patients with habitual cocaine use, this patient population may be at significantly increased risk for severe, invasive infections, despite the typically transient nature of neutropenia following levamisole ingestion.
